# Patients with Gastric Polyps need Colonoscopy Screening at Younger Age: A Large Prospective Cross-Sectional Study in China

**DOI:** 10.7150/jca.32857

**Published:** 2019-08-07

**Authors:** Shenghong Zhang, Danping Zheng, Zhiwei Yang, Liru Hong, Siew Chien Ng, Minrui Li, Shanshan Huang, Shengbing Wang, Li Li, Manying Li, Hongshi Zhang, Jinghua Lin, Bihui Zhong, Yi Cui, Minhu Chen

**Affiliations:** 1 Division of Gastroenterology, The First Affiliated Hospital, Sun Yat-sen University, Guangzhou, P.R. China; 2 Division of Gastroenterology, Meizhou People's Hospital, Meizhou, P.R. China; 3 Division of Gastroenterology, Shantou Central Hospital, Shantou, P.R. China; 4 Department of Medicine and Therapeutics, State Key Laboratory of Digestive Disease, Institute of Digestive Disease, Li Ka Shing Institute of Health Science, Hong Kong, P.R. China

**Keywords:** gastric polyp, colonoscopy, colorectal adenoma, colorectal cancer

## Abstract

**Background** To date, it is not clarified whether patients with gastric polyps without any alarming symptoms for colorectal neoplasia need colonoscopy screening. The objective of this study is to prospectively determine the association between gastric polyps and colorectal neoplasia.

**Methods** A multicenter prospective cross-sectional study was performed from July 2012 to December 2014. We compared patients with and without gastric polyps for prevalence of colorectal adenomas. The odds ratios (OR) were computed by logistic regression analysis after multivariable adjustments.

**Results** Totally 1546 patients were included, with 770 patients in the gastric polyp group and 776 in the age- and sex- matched control group. Patients with gastric polyps had greater odds of having any colorectal adenoma (adjusted OR=2.34, 95% confidence interval [CI]: 1.79 to 3.06, p<0.001) and advanced colorectal adenomas (adjusted OR=2.71, 95% CI: 1.74 to 4.23, p<0.001) than those without. The positive association between gastric polyps and colorectal adenomas remained significant in both women (OR=2.34, 95% CI: 1.66 to 3.29, p<0.001) and men (OR=1.87, 95% CI: 1.31 to 2.66, p=0.001). Patients over the age of 40 with gastric polyps had a higher prevalence of colorectal adenomas than those without (40-49yr: OR=1.81, 95% CI=1.02-3.21, p=0.04; 50-59yr: OR=1.88, 95% CI=1.26-2.81, p<0.001; 60-74yr: OR=2.62, 95% CI=1.73-3.98, p<0.001).

**Conclusions** The presence of gastric polyps is significantly associated with a higher prevalence of colorectal adenomas, especially advanced colorectal adenomas. Colonoscopy might be considered in patients with gastric polyps, of any gender, and over the age of 40.

## Background

Colonoscopy is widely accepted as a primary tool in screening for colorectal cancer (CRC) and precancerous polyps 1, 2. Colonoscopic polypectomy is associated with a 53% reduction in the long-term mortality of CRC 3. Current national guidelines recommend CRC screening in average-risk asymptomatic individuals aged more than 50 to prevent cancer incidence and mortality 4, 5. In countries with limited resources, it is important to risk-stratify patients for CRC screening. Risk factors for CRC and advanced adenomas have been identified, including age, sex, smoking, and family history 6. Additionally, colorectal neoplasms occur more frequently in patients with gastric cancer 7, 8. However, detection of colorectal neoplasms in gastric cancer patients might not change the prognosis greatly, especially in late stage of cancer. Thus, it is important to know whether individuals with sporadic gastric polyps would also benefit from screening colonoscopy by detecting synchronous or metachronous colorectal neoplasms.

To date, available knowledge regarding this issue is limited and has been gathered in a meta-analysis.^9^ The combined analysis of five heterogeneous retrospective studies suggested that the prevalence of colorectal neoplasms was 1.15 to 1.31 times higher in patients with gastric polyps than those without, focusing mainly on fundic gland polyps (FGP) 9. However, these retrospective studies were unable to differentiate various confounding factors that influenced the association. Selection bias was inevitably introduced, making the conclusions less convincing. Moreover, whether the associations between gastric polyps and colorectal adenomas differ by age and sex stratifications is not clarified yet. Although one study has shown that the association between gastric polyps and colorectal polyps remained positive after gender stratification 10, another study only found a positive association in women 11. Besides, very few studies addressed the outcome of advanced colorectal adenomas, despite only one case-control study with small sample size 12, showing no significant association between gastric polyps and the advanced colorectal adenomas.

Therefore, the current evidence regarding the association between sporadic gastric polyps and colorectal neoplasia remains scarce, mainly based on restricted-sized retrospective studies. To address this issue, we conducted a large multicenter prospective cross-sectional study to investigate the potential relationship between the presence of gastric polyps and prevalence of colorectal adenomas.

## Methods

### Study design and participants

We performed a cross-sectional study (ChiCTR--OCC-12003010) by matching at-risk subjects (those with gastric polyps) and disease-free subjects (those without gastric polyps) and determining the likelihood that these patients had colorectal adenomas, including advanced colorectal adenomas. This multicenter study was conducted in the First Affiliated Hospital of Sun Yat-sen University, Shantou Central Hospital, and Meizhou People's Hospital. Consecutive subjects who underwent esophagogastroduodenoscopy (EGD) were prospectively enrolled between July 2012 and December 2014. Participants in the gastric polyp group had different types of polyps, detected by EGD and confirmed by pathology, including FGP, gastric hyperplastic polyps, adenomas, and inflammatory polyps. The control group comprised of sex- and age-matched participants without detection of gastric polyps by EGD during the same period, randomly selected using a one-to-one matching approach. Both groups of patients underwent total colonoscopy at the same day or within six months of the EGD procedure. Participants were included if they were between the ages of 18 and 74, and excluded according to the following criteria: 1) having a history of gastrointestinal tumors or colorectal polyps; 2) having a history of gastrointestinal surgery, including colorectal polypectomy; 3) having a history of inflammatory bowel disease, intestinal tuberculosis, or familial colorectal polyposis syndrome; 4) presenting with alarm symptoms of CRC, including hematochezia, positive fecal occult blood test, recent bowel habit changes, significant weight loss, or anemia; 5) failing to undergo colonoscopy within six months after EGD; 6) technically unsatisfactory colonoscopy owing to poor or inadequate bowel preparation or incomplete colonoscopy; and 7) with gastric polyps that were histologically confirmed as carcinoid tumors, lymphomas, fibromas, lipomas, leiomyomas, ectopic pancreatic tissues, or normal tissue.

The study was conducted and reported according to the study protocol, conforming to the ethical guidelines of the 1975 Declaration of Helsinki, which was approved by Ethics Review Committees at each study center on 28 June, 2012. Written informed consents were obtained from participants upon their arrival at the study clinic and before the procedures.

### Endoscopic procedure and pathological examinations

At each participating center, EGD and colonoscopy procedures were performed by experienced gastroscopists and colonoscopists respectively, who had performed at least 2000 gastroscopies, 1000 colonoscopies, and 200 polypectomies. Patients were prescribed two liters of polyethylene glycol oral lavage for bowel preparation 4 to 6 hours before the colonoscopy procedure. All polyps detected during EGD and colonoscopy were recorded, including the number, size, and location. Polyp size was estimated endoscopically using open biopsy forceps, or measured following specimen resection. Gastric polyps were classified according to their location in the fundus, body, antrum, cardia, or pylorus. The locations of colorectal polyps were categorized as right colon (proximal to splenic flexure or a colonoscopic insertion depth of more than 55 cm), left colon (distal to splenic flexure or an insertion depth of less than 55 cm), or both right and left colon, as previously reported 13. The pathology of all resected colorectal polyps was evaluated by certified gastrointestinal pathologists, classified as non-neoplastic (hyperplastic or inflammatory polyps) or neoplastic (adenomatous). Adenomatous polyps were categorized as tubular, tubulovillous, or villous adenomas according to the World Health Organization criteria 14. Gastroscopists, colonoscopists and pathologists were blinded to each other's findings.

### Outcome and variable assessment

The primary outcome was the association between the presence of gastric polyps and the prevalence of colorectal adenomas. The secondary outcome was the prevalence of advanced colorectal adenomas among subjects with gastric polyps compared to those without. We also sought to determine whether these associations differed when patients were stratified by sex and age. Patients with advanced adenomas were classified as having at least one adenoma with at least one of the following characteristics: size of ≥10 mm in diameter, tubulovillous or villous components, or high-grade dysplasia 15. Pathological diagnosis of intramucosal carcinoma or carcinoma in situ was classified as high-grade dysplasia 16. CRC was graded according to the TNM staging system 17.

Data regarding baseline characteristics of the participants were collected, including age, sex, body mass index (BMI), educational level, symptoms, family history of CRC, current smoking (≥1 cigarette per day for more than one year), alcohol consumption (at least 70 g per week), non-steroidal anti-inflammatory drugs (NSAIDS)/aspirin use (for at least three months) 18, history of hypertension (systolic pressure≥140 mmHg and/or diastolic≥90 mmHg), fasting hyperglycemia (≥7.0 mmol/L), hepatic steatosis (detected by abdominal ultrasonography), and dyslipidemia. Blood glucose and lipid profiles were measured following eight hours of fasting. *Helicobacter pylori* (*H. pylori*) infection was checked by urea breath tests or urease tests.

### Statistical analysis

Symmetrically distributed continuous variables were presented as means with standard deviations, while categorical variables were presented as frequencies and percentages. To compare the baseline characteristics of participants in each group, we used the Pearson's chi-squared test for analysis of discrete variables and a t-test for continuous variables. We examined the association between gastric polyps and colorectal adenomas using binary logistic regression analysis. Adjusted odds ratios (OR) and their corresponding 95% confidence intervals (CI) were estimated by multivariable adjustments of baseline factors. In addition, we evaluated the relationship between gastric polyps and colorectal adenomas according to the number, size, location, and histopathology of both gastric polyps and colorectal adenomas, using both unadjusted and adjusted ORs and their corresponding 95% CIs. We also compared the presence of colorectal adenomas in patients with and without gastric polyps after stratifying by age and sex.

Pre-specified subgroup analysis was performed based on baseline covariates. Whether the prevalence of colorectal adenoma differed in certain pre-specified subgroups was assessed by testing the interaction effect between gastric polyps and subgroups with the use of logistic models.

All statistical analyses were performed using the Statistical Product and Service Solutions (SPSS) software, version 22.0 (IBM Corp, Armonk, NY, USA). All two-tailed tests were regarded as significant with a p value of less than 0.05.

## Results

### Baseline characteristics of the study

**Figure 1:**
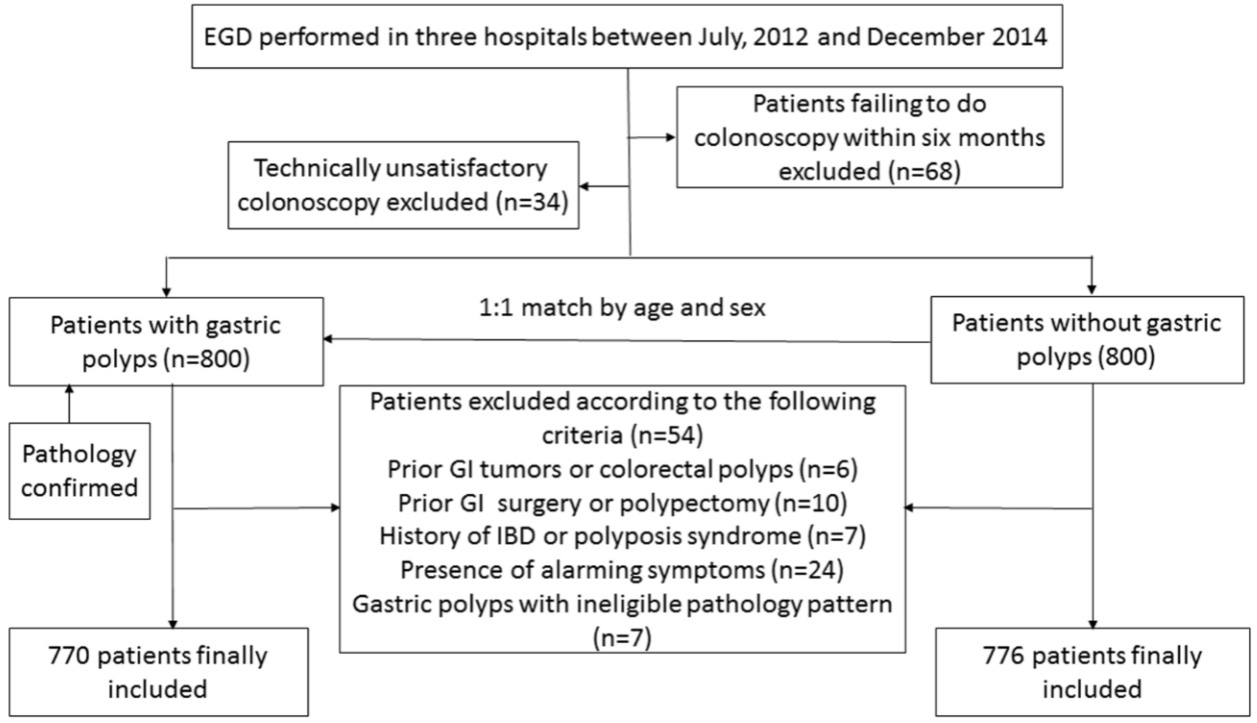
** Diagram of enrollment in study groups.** EGD: esophagogastroduodenoscopy; GI: gastrointestinal; IBD: inflammatory bowel disease.

A detailed participant enrollment process is presented in** Figure 1**. After excluding patients who failed to undergo colonoscopy within six months after EGD and patients with a technically unsatisfactory colonoscopy, 800 subjects with different types of gastric polyps, detected by EGD and confirmed by pathology, were enrolled according to the inclusion criteria. During the same time period, 800 sex- and age-matched gastric polyp-free control patients were enrolled. The indications for EGD included abdominal pain, gastroesophageal reflux, dyspepsia, upper gastrointestinal bleeding, and screening. After the application of strict exclusions, 1546 individuals were finally included, with 770 in the gastric polyp group and 776 in the gastric polyp-free control group.

Baseline characteristics were compared between patients with and without gastric polyps and the results were shown in **Table 1**. Subjects in these two groups did not differ significantly in age, sex, BMI, education level, positive CRC family history, hypertension, fasting hyperglycemia, steatosis, or potential use of chemopreventive agents, including NSAIDS and aspirin. However, significantly fewer subjects in the gastric polyp group were symptomatic (including abdominal pain, dyspepsia, and gastroesophageal reflux), had current smoking, alcohol consumption, and positive *H. pylori* infection than those in the gastric polyp-free group. Lipid profiles differed significantly between the two groups, except for triglyceride levels.

### Association of gastric polyps with colorectal adenomas

During the study period, we detected colorectal polyps in 506 participants by colonoscopy, including 198 of the 776 patients (25.5%) who did not have gastric polyps, and 308 of the 770 patients (40.0%) who did. Among the control group, we identified 133 subjects (17.1%) with any type of colorectal adenoma, of which 35 (4.5%) were classified as having advanced colorectal adenomas. In the gastric polyp group, 229 subjects (29.7%) had any type of colorectal adenoma, of which 77 (10.0%) had advanced colorectal adenomas. **Table 2** presents the ORs of gastric polyps and baseline factors for colorectal adenomas as well as advanced colorectal adenomas. After multivariable adjustments, we found that subjects with gastric polyps had greater odds of having any colorectal adenoma (adjusted OR=2.34, 95% CI: 1.79 to 3.06, p<0.001) as well as advanced colorectal adenomas (adjusted OR=2.71, 95% CI: 1.74 to 4.23, p<0.001) compared with those in the control group. The adjusted ORs of gastric polyps for colorectal adenomas in the First Affiliated Hospital of Sun Yat-sen University, Shantou Central Hospital, and Meizhou People's Hospital were 3.56 (95% CI: 2.41 to 5.26, p<0.001), 2.35 (95% CI: 1.03 to 5.37, p=0.04) and 2.65 (95% CI: 1.08 to 6.53, p=0.03), respectively.

### Association of gastric polyps with different types of colorectal neoplasia

Compared with the control group, patients with gastric polyps had significantly increased prevalence of colorectal adenomas when colorectal adenomas were stratified by number, location, size, and histopathology (**Table 3**). Besides adenomas, we found no difference in the prevalence of colorectal hyperplastic polyps (adjusted OR: 0.99, 95% CI: 0.67 to 1.47, p= 0.96) between patients with and without gastric polyps. Furthermore, we found that the prevalence of CRC was 1.3% (10/776) in the gastric polyp group and 0.3% (2/770) in the control group, with an adjusted OR of 7.05 (95% CI: 1.35 to 36.81, p=0.02) (Detailed characteristics of patients with CRC were shown in Supplemental Table 1). In the gastric polyp group, six early-stage adenocarcinomas were detected and removed endoscopically. Additionally, we identified serrated adenomas in one patient (1/776, 0.1%) in the control group and two patients (2/770, 0.3%) in the gastric polyp group.

### Association between gastric polyp characteristics and colorectal adenomas

Patients with gastric polyps had significantly higher prevalence of colorectal adenoma and advanced adenomas, when stratified by the number, distribution, size, histopathology of gastric polyps, and whether *H. pylori* infection was detected (**Table 4**). However, we found no significant correlation between gastric inflammatory polyps and the detection of advanced colorectal adenomas (adjusted OR=0.79, 95% CI: 0.24 to 2.64, p= 0.49).

### Association between gastric polyps and colorectal adenomas stratified by sex and age

Women with gastric polyps had higher prevalence of any colorectal adenoma (OR=2.34, 95% CI: 1.66 to 3.29, p<0.001) as well as advanced colorectal adenomas (OR=4.31, 95% CI: 2.05 to 9.02, p<0.001) compared with those without gastric polyps. Such significant associations remained in any type of colorectal neoplasia when FGP and gastric hyperplastic polyps were analyzed separately (**Table 5**). For men, subjects with gastric polyps had higher prevalence of any colorectal adenoma (OR=1.87, 95% CI: 1.31 to 2.66, p= 0.001) and advanced colorectal adenomas (OR=1.71, 95% CI: 1.01 to 2.88, p=0.04). However, the associations of gastric polyps with CRC were not significant in men (OR=1.57, 95% CI: 0.26 to 9.49, p=0.62), mainly due to limited cases of CRC. Furthermore, we found no significant association between gastric hyperplastic polyps and the prevalence of any colorectal adenoma type in men (**Table 5**).

Patients over the age of 40 with gastric polyps were associated with a higher prevalence of any adenoma (40-49yr: OR=1.81, 95% CI=1.02-3.21, p=0.04; 50-59yr: OR=1.88, 95% CI=1.26-2.81, p<0.001; 60-74yr: OR=2.62, 95% CI=1.73-3.98, p<0.001) and advanced adenomas (40-49yr: OR=2.87, 95% CI=1.10-7.45, p=0.03; 50-59yr: OR=1.93, 95% CI=0.98-3.79, p=0.05; 60-74yr: OR=2.17, 95% CI=1.11-4.23, p=0.02). The association remained significant in patients with FGP. In patients with gastric hyperplastic polyps over the age of 50, we detected a significantly higher rate of colorectal adenomas. In subjects under 40 years of age, no significant associations between gastric polyps and colorectal adenomas were detected (**Table 6**).

### Subgroup analysis

The increase in colorectal adenoma prevalence in patients with gastric polyps was accordant across all major subgroups based on baseline factors (**Supplemental Figure 1**). Subgroup analysis suggested no significant interactions in any of the 17 predefined subgroups (P> 0.05 for all comparisons).

## Discussion

The current study prospectively analyzed 1546 consecutive patients and confirmed an association between gastric polyps and colorectal adenomas, especially advanced colorectal adenomas. A significantly higher prevalence of colorectal adenomas (29.7%) and advanced colorectal adenomas (10.0%) were observed in patients with gastric polyps compared to the matched control group (17.1% for colorectal adenomas and 4.5% for colorectal adenomas). Both men and women with gastric polyps had significantly higher prevalence of colorectal adenomas than those of control groups. Furthermore, Subjects over the age of 40 with gastric polyps were more likely to have both advanced and malignant colorectal adenomas.

Although previous studies have shown that FGP 19, 20, hyperplastic gastric polyps 21 and gastric adenomas 10, 22 are associated with a higher prevalence of colorectal polyps, most of these were based on retrospective design and limited sample size. The higher prevalence of colorectal adenomas and advanced colorectal adenomas in our cohort is backed up by a multicenter prospective cross-sectional design with large sample size, and particularly, by comprehensive multivariable adjustments and detailed stratification analysis. It should be noted that in this study, patients with gastric polyps had less smoking and alcohol consumption and higher serum cholesterol levels at baseline. This might be due to the reason that we included only age- and sex- matched subjects, which could possibly limit the balance of these baseline risk factors. We also noticed lower *H. pylori* infection rate in the gastric-polyp group at baseline. It is known that *H. pylori* infection are negatively associated with FGP 23 but positively associated with gastric hyperplastic polyps 24. In this study, we included subjects with different kinds of gastric polyps, with 66% being FGP, while only 21% being hyperplastic polyps, thus can explain the lower *H. pylori* infection rate in the gastric-polyp group. Despite this, strong positive correlation between gastric polyps and colorectal adenomas still exists after adjusting the confounding effects of these factors.

The finding that higher prevalence of colorectal adenomas among patients with gastric polyps in this study are of clinical significance, because of the malignancy potential of colorectal adenomas 25. The majority of CRCs derive from neoplastic adenomatous polyps of the mucosa, among which advanced adenomas are regarded as the clinically relevant precursors 15. Early removal of these polyps before their progression to malignancy can substantially reduce the subsequent incidence and mortality of CRC 3, 26, 27. The prevalence of CRC is significantly higher among patients with gastric polyps compared with the control group, which is consistent with a previous study 19. Importantly, 60% of all colonic adenocarcinomas detected in the gastric polyp group were graded as early-stage cancer and could be endoscopically removed. Early diagnosis of CRC, when tumors are still localized, permits simpler treatment by endoscopic polypectomy or open surgery, which are strongly supported by current guidelines 2, 4, 5. Based on these results, we highly recommend colonoscopy screening among patients with gastric polyps without alarming symptoms.

Male gender is a recognized risk factor for colorectal neoplasia 28. We confirmed this finding and found that the presence of gastric polyps in male subjects further raised the prevalence of colorectal adenomas. Another large-scale retrospective study from 2009 demonstrated a higher prevalence of colorectal adenomas in women who had FGP (OR=1.43, 95% CI: 1.26-1.63) than in men 11. Here, we found a significant correlation between gastric polyps and colorectal adenomas in both women and men, not only for FGP, but also for all types of gastric polyps, although the risks for women were higher than for men. Women with gastric polyps were more likely to have advanced and malignant colorectal adenomas than men, which was another interesting finding. These results demonstrate that the presence of gastric polyps, either FGPs or gastric hyperplastic polyps, can greatly increase the prevalence of colorectal adenomas among women. Therefore, we also recommend that women with gastric polyps undergo colonoscopy for screening of cancer precursor lesions.

The risk of colorectal neoplasia increases with age 28, 29, and the current guidelines recommend colonoscopy screening in average-risk patients over the age of 50 4. Interestingly, patients over 40 with gastric polyps were more likely to have colorectal adenomas than younger patients. Among patients younger than 40 with gastric polyps, prevalence of colorectal adenomas is not significantly increased. Although patients over 50 are already candidates for colonoscopy screening, we recommend that patients with gastric polyps begin screening colonoscopy as early as 40, especially among patients with FGP, since a fair amount of colorectal neoplasms can be detected among this population during the 10-year gap.

The association between gastric and colorectal neoplasia is well clarified in hereditary cancer syndromes, such as familial adenomatous polyposis (FAP) 30 or hereditary non-polyposis colorectal cancer 31. However, the precise mechanism underlying the potential relationship between sporadic gastric polyps and colorectal adenomas remains unclear. It is suspected that both genetic and environmental factors play important roles. It has been reported that the genetic alterations found in sporadic gastric and colorectal adenomas are similar 32. For example, activation of β-Catenin gene mutations is demonstrated both in the pathogenesis of sporadic FGPs 33 and colorectal adenomas 34. Specific gene mutations, including adenomatous polyposis coli (APC), Kirsten-ras (K-RAS), and p53, are implicated in both colorectal and gastric tumorigenesis, by driving the progression from healthy epithelia to dysplasia and cancer 35, 36. Other types of genetic and epigenetic abnormalities, including microsatellite instability and hypermethylation have been associated with primary gastrointestinal cancers 37. Despite this, the genetic associations between gastric and colorectal polyps are not totally clarified yet, and implementation of next generation sequencing method is warranted to identify novel genetic and epigenetic alterations. The role of environmental factors underlying the gastric-colorectal polyps association remains largely unexplored. Although lifestyle factors such as smoking has been shown to be positively related to both colorectal polyps and gastric polyps 38-40, the mechanism of how these non-genetic factors contributes to the development of both gastric and colorectal polyps merits further studies. Alteration of gut microbiota has been shown to be associated with colorectal adenomas and gastric polyposis^41^, ^42^. However, controversial evidence has been presented regarding the association of *H. Pylori* infection and colonic neoplasia 43, 44. In our study, the association of gastric polys and colorectal adenomas exists irrespective of the status of H. pylori infection. Therefore, the interactions between H. pylori and other gastric bacteria and their roles in gastric-colorectal polyp co-occurrence remains unknown and merits further studies.

There were a few shortcomings of this study. First, colonoscopy was performed only within six months of EGD. Due to the prolonged formation and slow growing of polyps 4, it is very likely that colorectal polyps were present at the time of EGD. Thus, the risk of de novo colorectal polyp initiation among patients with gastric polyps remains unclear, and long-term follow up study is necessary. Second, although at least six minutes of withdrawal time was required for colonoscopy, we did not measure this. We were unable to eliminate the confounding effect from withdrawal time, since it has been confirmed to be associated with increased adenoma detection 45. Third, due to the very low detection rate of serrated polyps in this study, we were unable to assess the association between gastric polyps and the serrated colorectal polyps, a separate entity with malignant potential 46. Finally, despite the multicenter advantage of this study, given the geographic variation of colorectal polyps and CRCs among Western and Eastern countries, the generalizability of our results to other regions needs to be further confirmed.

## Conclusions

In summary, our results demonstrate that the presence of gastric polyps, regardless of their number, size, location, or pathology, is an independent risk factor for the detection of colorectal adenomas, particularly advanced adenomas. We recommend screening colonoscopy in patients of both sexes over the age of 40 with gastric polyps. Further studies are required to elucidate the initiation of de novo colorectal polyps among patients with gastric polyps, the cost effectiveness of such a colonoscopy screening approach, the application of our results to Western countries, and the clarification of the precise mechanism underlying the close relationship between gastric and colorectal polyps.

## Supplementary Material

Supplementary figures and tables.

## Figures and Tables

**Table 1:** Comparison of baseline characteristics between subjects with and without gastric polyps

Characteristics	Non-gastric polyps (n=776)	Gastric polyps (n=770)	P value
Age (years)	52.4±11.3	52.6±11.5	0.67
Sex (% male)	302 (38.9%)	289 (37.5%)	0.58
College graduate (%)	95 (12.2%)	93 (12.1%)	0.92
BMI≥25 kg/m^2^ (%)	130 (16.8%)	132 (17.1%)	0.84
Family history of CRCs (%)	27 (3.5%)	33 (4.3 %)	0.41
Current smoking (%)	103 (13.3%)	51 (6.6%)	<0.001
Alcohol consumption (%)	50 (6.4%)	22 (2.9%)	0.001
History of hypertension (%)	100 (12.9%)	115 (14.9%)	0.24
NSAIDS/aspirin use (%)	18 (2.3%)	22 (2.9%)	0.51
Presenting with symptoms (%)*	599 (77.2%)	543 (70.5%)	0.003
Presence of hepatic steatosis (%)†	117 (15.1%)	114 (14.8%)	0.88
Positive H. pylori infection (%)	369 (47.6%)	250 (32.5%)	<0.001
Lipid profiles and blood glucose			
Triglyceride (mmol/L)	1.31±1.15	1.35±0.96	0.45
Total cholesterol (mmol/L)	4.69±1.40	5.15±1.12	<0.001
LDL cholesterol (mmol/L)	2.56±0.96	3.03±0.96	<0.001
HDL cholesterol (mmol/L)	1.32±0.40	1.43±0.36	<0.001
Fasting hyperglycemia (≥7.0mmol/L) (%)	61 (7.9%)	42 (5.5%)	0.06

* Patients with alarm symptoms of CRC (including hematochezia, positive fecal occult blood test, recent bowel habit changes, significant weight loss, or anemia) were excluded from this study; patients with symptoms of abdominal pain, dyspepsia and gastroesophageal reflux were included. †The presence of hepatic steatosis was detected by abdominal ultrasonography. BMI-body mass index; CRC-colorectal cancer; H. pylori-helicobacter pylori; NSAIDS-non-steroidal anti-inflammatory drugs; LDL- low density lipoprotein; HDL-high density lipoprotein.

**Table 2:** Multivariable analyses on odds ratios of baseline characteristics for colorectal neoplasia

Variables	Any adenoma		Advanced adenoma	
	Multivariable analysis		Multivariable analysis	
	OR (95% CI)	P value	OR (95% CI)	P value
Male gender	1.65 (1.26,2.16)	<0.001	1.98 (1.28,3.05)	0.002
Age, year	1.04 (1.03,1.05)	<0.001	1.03 (1.01,1.05)	0.002
BMI, kg/m^2^	1.02 (0.98,1.07)	0.36	1.05 (0.98,1.13)	0.17
College graduate	0.88 (0.59,1.31)	0.52	0.55 (0.26,1.17)	0.12
CRC Family history	1.94 (1.06,3.54)	0.03	2.47 (1.08,5.66)	0.03
Current smoking	2.33 (1.52,3.58)	<0.001	1.97 (1.06,3.67)	0.03
Alcohol consumption	1.08 (0.60,1.95)	0.80	1.16 (0.49,2.75)	0.74
History of hypertension	1.44 (1.02,2.03)	0.04	0.84 (0.47,1.50)	0.56
NSAIDS/aspirin use	0.44 (0.19,1.02)	0.06	0.61 (0.17, 2.17)	0.45
Symptomatic	0.73 (0.55,0.97)	0.03	0.77 (0.50,1.19)	0.24
Presence of steatosis	1.17 (0.81,1.69)	0.40	0.91 (0.50,1.63)	0.74
Positive H. pylori infection	1.19 (0.92,1.55)	0.19	0.92 (0.60, 1.40)	0.69
Fasting glycemia≥7.0mmol/L	1.06 (0.65,1.72)	0.83	1.52 (0.75,3.05)	0.25
Triglyceride>1.70 mmol/L	0.88 (0.63,1.23)	0.46	1.09 (0.65,1.82)	0.75
Total cholesterol>5.70 mmol/L	1.20 (0.81,1.77)	0.36	1.01 (0.54,1.89)	0.98
LDL>3.61 mmol/L	1.17 (0.79,1.74)	0.43	0.79 (0.42,1.50)	0.47
HDL<1.09 mmol/L	1.31 (0.95,1.82)	0.10	1.63 (1.00,2.65)	0.05
Gastric polyps	**2.34 (1.79,3.06)**	**<0.001**	**2.71 (1.74,4.23)**	**<0.001**

BMI-body mass index; CRC-colorectal cancer; H. pylori-helicobacter pylori; NSAIDS-non-steroidal anti-inflammatory drugs; LDL- low density lipoprotein; HDL-high density lipoprotein; OR-odds ratio; CI-confidence interval.

**Table 3:** Unadjusted and adjusted odds ratios for the prevalence of colorectal neoplasia stratified by different types

Characteristics of colorectal polyps	No. in non-gastric polyp group (%)	No. in gastric polyp group (%)	Unadjusted		Adjusted	
			OR (95% CI)	P value	OR (95%CI)	P value
Adenoma						
Number						
Single	62 (8.0%)	111(14.4%)	1.94(1.40,2,69)	<0.001	1.91(1.37,2.66)	<0.001
Multiple (≥2)	71(9.1%)	118(15.3%)	1.80(1.31,2,46)	<0.001	2.16(1.54,3.01)	<0.001
Distribution						
Right colon	36(4.6%)	88(11.4%)	2.65(1.78,3.96)	<0.001	2.61(1.74,3.92)	<0.001
Left colon	57(7.3%)	147(19.1%)	2.98(2.15,4.12)	<0.001	3.16(2.27,4.39)	<0.001
Both	40(5.2%)	73(9.5%)	1.93(1.29,2.87)	0.001	1.95(1,29,2.93)	0.001
Largest polyp size						
<5mm	36 (4.6%)	59 (7.7%)	1.71(1.11, 2.62)	0.013	1.68(1,08,2.62)	0.021
5-9mm	68(8.4%)	104(13.5%)	1.71(1.23, 2.37)	0.001	1.76(1,26,2.47)	0.001
≥10mm	32(4.1%)	66(8.6%)	2.18(1.41, 3.37)	<0.001	2.37(1,52,3.70)	<0.001
Histology (other than advanced adenoma)						
Tubular adenoma	118(15.2%)	203(26.4%)	2.00 (1.55, 2.57)	<0.001	2.17(1.65,2.85)	<0.001
Tubulovillous/villous adenoma	21(2.7%)	40(5.2%)	1.97(1.15, 3.37)	0.012	1.82(1.03,3.21)	0.04
Serrated adenoma	1 (0.1%)	2(0.3%)	--	--	--	--
CRC	2 (0.3%)	10 (1.3%)	5.61(1.24, 25.39)	0.01	7.05(1.35,36.81)	0.02
Non-neoplastic polyps						
Hyperplastic polyps	60(7.7%)	59(7.7%)	0.99(0.68,1.44)	0.96	0.99(0.67,1.47)	0.96
Inflammatory polyps	11(1.4%)	40(5.2%)	3.81(1.94,7.48)	<0.001	4.19(2.08,8.42)	<0.001

OR-odds ratio; CI-confidence interval; CRC-colorectal cancer.

**Table 4:** Adjusted odds ratios for the detection of colorectal neoplasia stratified by different characteristics of gastric polyps

Subgroup		Advanced adenoma		Any adenoma		
	No. (%)	Adjusted OR (95% CI)	P value	No. (%)	Adjusted OR (95% CI)	P value
Non-gastric polyps (n=776)	35 (4.5%)	1.00		133(17.1%)	1.00	
Gastric polyps						
Histology pattern						
FGP (n=513)	54(10.5%)	2.89 (1.82,4.58)	<0.001	160(31.2%)	2.45 (1.85,3.24)	<0.001
Hyperplastic polyps(n=166)	20(12.0%)	3.16 (1.72,5.83)	<0.001	43 (25.9%)	1.82 (1.21,2.77)	0.004
Adenomatous polyps(n=11)	3 (27.3%)	7.94(2.02,31.23)	0.013	5 (45.5%)	4.03 (1.21,13.40)	0.029
Inflammatory polyps(n=83)	3 (3.6%)	0.79(0.24,2.64)	0.49	25 (30.1%)	2.53 (1.49,4.29)	0.001
Number						
Single (n=418)	39 (9.3%)	2.89 (1.82,4.58)	<0.001	125(29.9%)	2.44 (1.80,3.31)	<0.001
Multiple (n=352)	54(10.5%)	2.89 (1.82,4.58)	<0.001	104(29.5%)	2.24 (1.64,3.07)	<0.001
Size						
<0.5cm (n=451)	40 (8.9%)	2.14 (1.32,3.48)	<0.001	133(29.5%)	2.12 (1.59,2.84)	<0.001
≧0.5cm (n=319)	54(10.5%)	2.89 (1.82,4.58)	<0.001	96 (30.1%)	2.42 (1.75,3.35)	<0.001
Location						
Gastric body (n=401)	40(10.0%)	2.81 (1.71,4.61)	<0.001	120(29.9%)	2.33 (1.72,3.15)	<0.001
Fundus (n=386)	38 (9.8%)	2.45 (1.51,3.98)	<0.001	120(31.1%)	2.39 (1.77,3.22)	<0.001
Antrum (n=108)	12(11.1%)	2.99 (1.42,6.27)	0.004	28 (25.9%)	1.94 (1.18,3.17)	0.009
Others (n=55) ^#^	7 (12.7%)	4.26 (1.71,10.67)	0.002	14 (25.5%)	1.95 (1.01,3.78)	0.047
H. pylori infection						
Negative (n=520)	53(10.2%)	2.81 (1.77,4.46)	<0.001	147(28.3%)	2.26 (1.69,3.01)	<0.001
Positive (n=250)	24 (9.6%)	2.40 (1.38,4.18)	0.002	14 (25.5%)	2.54 (1.80,3.57)	<0.001

# including cardia, pylorus and angle. FGP: fundic gastric polyp.

**Table 5:** Risk of colorectal adenomas in groups with and without gastric polyps according to sex.

colorectal neoplasia		Group with all gastric polyps			Group with fundic gland polyps			Group with hyperplastic polyps	
		OR (95% CI)	P value		OR (95% CI)		P value	OR (95% CI)	P value
Any adenoma									
Men		1.87(1.31,2.66)	0.001		2.11(1.43,3.11)		<0.001	1.28(0.72,2.30)	0.40
Women		2.34(1.66,3.29)	<0.001		2.41(1.67,3.50)		<0.001	2.16(1.26,3.71)	0.005
Advanced adenoma									
Men		1.71(1.01,2.88)	0.04		2.04(1.17,3.57)		0.01	1.42(0.61,3.28)	0.42
Women		4.31(2.05,9.02)	<0.001		3.99(1.82,8.74)		<0.001	7.21(2.95,17.63)	<0.001
Tubular adenoma									
Men		1.83(1.26,2.65)	0.001		1.99(1.32,2.99)		0.001	1.37(0.75,2.50)	0.31
Women		2.32(1.57,3.18)	<0.001		2.32(1.59,3.40)		<0.001	2.08(1.19,3.63)	0.01
Tubulovillous/villous adenoma									
Men		1.25(0.64,2.43)	0.52		1.63(0.81,3.27)		0.17	0.51(0.12,2.25)	0.36
Women		5.10(1.73,15.03)	0.001		5.76(1.89,17.52)		<0.001	5.00(1.23,20.35)	0.01
CRC									
Men		1.57(0.26,9.49)	0.62		1.58(2.22,11.30)		0.65	2.24 (0.2,25.05)	0.50
Women		1.02(1.01,1.03)	0.01		1.02(1.01,1.04)		0.001	1.01(0.99,1.03)	0.03
									

^#^ Number of colorectal polyps. OR-odds ratio; CI-confidence interval; CRC-colorectal cancer.

**Table 6:** Risk of colorectal adenomas in groups with and without gastric polyps according to different age stratifications.

Colorectal adenomas	Group with gastric polyps		Group with fundic gastric polyps		Group with hyperplastic gastric polyps	
	OR (95% CI)	P value	OR (95% CI)	P value	OR (95% CI)	P value
Any adenoma						
18-29 yr	3.65(0.38,35.34)	0.24	4.67(0.48,45.62)	0.35	7.00(0.34,144.06)	0.29
30-39 yr	1.44(0.60,3.48)	0.41	1.19(0.43,3.28)	0.80	1.42(0.28,7.27)	0.48
40-49 yr	1.81(1.02,3.21)	0.04	2.31(1.25,4.26)	0.01	1.27(0.48,3.38)	0.40
50-59 yr	1.88(1.26,2.81)	<0.001	2.13(1.38,3.31)	0.001	1.80(0.95,3.41)	0.05
60-74 yr	2.62(1.73,3.98)	<0.001	2.50(1.59,3.95)	<0.001	1.66(0.85,3.23)	0.10
Advanced adenoma						
18-29 yr	1.08(0.97,1.20)	0.50	1.10(0.96,1.26)	0.49	1.33(0.76,2.35)	0.15
30-39 yr	5.26(0.58,48.06)	0.17	5.76(0.58,56.81)	0.13	8.00(0.47,138.38)	0.22
40-49 yr	2.87(1.10,7.45)	0.03	3.24(1.18,8.89)	0.02	1.53(0.30,7.89)	0.40
50-59 yr	1.93(0.98,3.79)	0.05	2.33(1.14,4.76)	0.03	2.86(1.14,7.19)	0.04
60-74 yr	2.17(1.11,4.23)	0.02	1.89(0.90,3.96)	0.13	2.65(1.05,6.68)	0.05

OR-odds ratio; CI-confidence interval.

## Abbreviations

CRC: colorectal cancer
FGP: fundic gland polyp
EGD: esophagogastroduodenoscopy
BMI: body mass index
NSAIDS: non-steroidal anti-inflammatory drugs
OR: odds ratio
CI: confidence interval
