# Supplementary material for: Patients with Gastric Polyps need Colonoscopy Screening at Younger Age: A Large Prospective Cross-Sectional Study in China
Source: J Cancer. 2019 Aug 7;10(19):4623–32. doi: 10.7150/jca.32857 (PMC6746131; doi:10.7150/jca.32857)

Supplementary material for:

Patients with Gastric Polyps need Colonoscopy Screening at Younger Age: A Large Prospective Cross-Sectional Study in China

Methods

Sample size estimation

To predict an appropriate sample size, a pilot study was conducted that included 30 subjects with gastric polyps and another 30 age- and sex-matched subjects without gastric polyps. Eight patients with colorectal polyps were detected in the gastric polyp group, compared with six in the control group. Therefore, we used a prevalence of 0.27 for the gastric polyp group and 0.20 for the control group to calculate sample size. Considering that a maximum of 10% of patients might fail to undergo colonoscopy within six months after EGD, we needed at least 724 subjects per group to achieve at least 85% power at 5% significance. Power Analysis and Sample Size software (PASS, LLC, Kaysville, Utah, USA) was used to calculate the sample size.

Supplemental Table 1: Characteristics of 12 patients with colorectal cancers.

**Case no./enrollment year**	**Age/sex**	**BMI (kg/m2)**	**Family history of CRC**	**Esophagogastroduodenoscopy**		**Characteristic of polyps in colonoscopy and TNM staging**				**Treatment**
				Indication	Pathology	Size	Location	Pathology	TNM staging	
**Group with gastric polyps**										
**1/2012**	65/female	21.4	No	Epigastric pain	FGP	4.0cm	Descending colon	Adenocarcinoma	Stage Ⅰ T2N0M0	Surgery
**2/2012**	36/female	21.4	No	Epigastric pain	FGP	1.5cm	Descending colon	Adenocarcinoma	Stage Ⅰ T1N0M0	Endoscopically removed
**3/2012**	65/male	22.3	No	Heartburn	FGP	0.8cm	Rectum	Intramucosal adenocarcinoma	Stage Ⅰ TisN0M0	Endoscopically removed
**4/2013**	63/female	21.2	Yes	Screening	FGP	0.8cm	Rectum	Adenocarcinoma	Stage Ⅰ T1N0M0	Endoscopically removed
**5/2013**	57/male	22.5	No	Epigastric pain	FGP	4.0cm	Sigmoid colon	Adenocarcinoma	Stage Ⅰ T1N0M0	Endoscopically removed
**6/2013**	68/male	18.1	No	Epigastric pain	Hyperplastic polyp	1.5cm	Sigmoid colon	Adenocarcinoma in situ	Stage 0 TisN0M0	Endoscopically removed
**7/2013**	68/female	24.4	No	Screening	Hyperplastic polyp	2.0cm	Sigmoid colon	Intramucosal adenocarcinoma	Stage 0 TisN0M0	Endoscopically removed

**8/2013**	51/male	23.6	No	Epigastric pain	FGP	Surrounding the lumen	Rectum-sigmoid junction	Adenocarcinoma	Stage Ⅰ T2N0M0	Surgery
**9/2013**	66/female	25.7	No	Screening	FGP	1.5cm	Sigmoid colon	Adenocarcinoma	Stage Ⅳ T2NxM1[#]	Palliative treatment
**10/2014**	44/female	27.5	No	Screening	FGP	0.4cm	Transverse colon	Adenocarcinoma	Stage Ⅰ T2N0M0	Surgery
**Group without gastric polyps**										
**12/2012**	70/male	15.2	No	Melena	Duodenal ulcer	0.8cm	Sigmoid colon	Adenocarcinoma	Stage Ⅰ T1N0M0	Endoscopically removed
**13/2012**	64/male	24.8	No	Screening	Chronic gastritis	2.0cm	Rectum	Adenocarcinoma	Stage Ⅰ T1N0M0	Endoscopically removed

BMI-body mass index; CRC-colorectal cancer; FGP-fundic gland polys

TNM staging: T-primary tumor, Tis-carcinoma in situ, intraepithelial or invasion of lamina propria, T1-tumor invades submucosa, T2-tumor invades muscularis propria; N- regional lymph nodes, Nx-regional lymph nodes cannot be assessed, N0-no regional lymph node metastasis; M- distant metastasis, M0-no distant metastasis, M1-distant metastasis ([#]liver metastasis in case no. 9).

Supplemental Figure 1. Odds Ratio for detection of colorectal adenomas in pre-specified subgroups.

*the unit for body mass index is kg/m^2, #the unit for blood glucose and lipid level is mmol/L.

NSAIDS-non-steroidal anti-inflammatory drugs; LDL-low density lipoprotein; HDL-high density lipoprotein.

Subgroup	No. of Patients	Non-gastric Polyps	Gastric Polyps	Odds Ratio (95% CI)	P Value
		no.of colorectal adenomas(%)			
Overall	1546	133(17.1%)	229(29.7%)	1.93（1.55,2.39）	
Sex					0.37
Male	591	74(24.5%)	109(37.7%)	1.87（1.31,2.66）	
Female	955	59(12.4%)	120(24.9%)	2.34（1.66,3.29）	
Age					0.42
<50 yr	394	34(11.5%)	54(18.7%)	1.76（1. 11,2.81）	
≧50yr	1152	99(20.6%)	175(36.4%)	2.21（1.65,2.95）	
Body mass index*					0.54
<25	1285	108(16.7%)	181(28.4%)	1.97（1.51,2.58）	
≧25	261	25(19.2%)	48(36.4%)	2.41（1.37,4.21）	
College gradute					0.97
No	1356	118(17.3%)	203(30.0%)	2.04（1.58,2.64）	
Yes	190	15(15.8%)	26(28.0%)	2.07（1.01,4.23）	
Family history					0.65
No	1482	127(17.0%)	214(29.2%)	2.01（1.57,2.58）	
Yes	64	6(21.4%)	15(41.7%)	2.62（0.86,8.03）	
Hypertention					0.44
No	1331	105(15.5%)	184(28.1%)	2.12（1.62,2.78）	
Yes	215	28(28.0%)	45(39.1%)	1.65（0.93,2.94）	
Glycemia≧7.0#					0.50
No	1444	117(16.4%)	214(29.4%)	2.13（1.65,2.74）	
Yes	102	16(26.2%)	15(35.7%)	1.56（0.67,3.66）	
Smoking					0.74
No	1374	95(14.2%)	196(27.7%)	2.31（1.76,3.03）	
Yes	172	38(34.9%)	33(52.4%)	2.06（1.09,3.87）	
Alcohol					0.29
No	1475	119(16.4%)	216(28.9%)	2.07（1.61,2.67）	
Yes	71	14(28.0%)	13(59.1%)	3.71（1.30,10.62）	
Steatosis					0.48
No	1316	164(24.9%)	251(38.2%)	1.87（1.47,2.36）	
Yes	230	34(29.1%)	55(48.7%)	2.32（1.34,3.99）	
Hp infection					0.90
No	928	63(15.5%)	147(28.3%)	2.15（1.55,2.99）	
Yes	618	70(19.0%)	82(32.8%)	2.09（1.44,3.02）	
NSAIDS use					0.68
No	1506	130(17.2%)	224(29.9%)	2.07（1.62,2.64）	
Yes	40	3(16.7%)	5(22.7%)	1.47（0.30,7.22）	
Triglyceride>1.70#					0.84
No	1243	105(16.5%)	171(28.3%)	2.00（1.52,2.63）	
Yes	303	28(20.3%)	58(35.2%)	2.13（1.26,3.60）	
Cholesterol>5.70#					0.89
No	1232	106(16.3%)	160(27.6%)	1.96（1.49,2.59）	
Yes	314	27(21.8%)	69(36.3%)	2.05（1.22,3.44）	
LDL>3.61#					0.39
No	1268	112(16.6%)	159(26.9%)	1.85（1.41,2.43）	
Yes	278	21(21.0%)	70(39.3%)	2.44（1.38,4.30）	
HDL<1.09#					0.59
No	1235	91(15.8%)	184(28.0%)	2.08（1.57,2.76）	
Yes	311	42(21.1%)	45(39.5%)	2.44（1.47,4.05）	
Symptom					
No	404	34(19.2%)	84(37.0%)	2.47（1.56,3.92）	0.52
Yes	1142	99(16.5%)	145(26.7%)	1.84（1.38,2.45）	

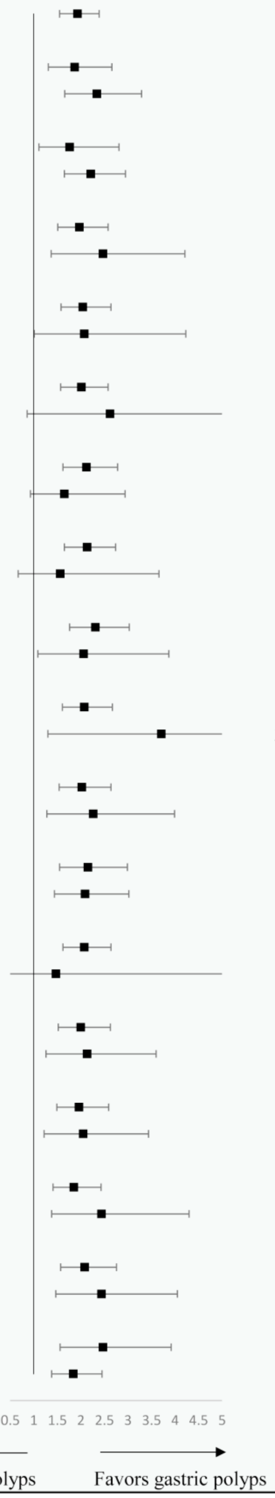

Supplement: Supplementary file 1 — Supplementary figures and tables. [file jcav10p4623s1.pdf]
